# The Making of American Doctors

**Published:** 1894-01-13

**Authors:** 


### Studies in Applied Sociology.

### II.—THE MAKING OF AMERICAN DOCTORS.

There are one hundred and twenty-nine medical colleges in the United States, besides twenty-seven schools of dentistry. To give any general idea of these is impossible, for they vary from nearly the best to the worst possible. European graduates sometimes go to study at the New York Post Graduate School, while there be schools to whose graduates one would not entrust the care of a cat—unless one wished to dock the number of the cat's nine lives.

It is easy to found a University in America. There was once (and that not ten years ago) a man who got from the Legislature of his State permission to found a University. He thereupon entitled his house a College, created himself LL.D., Principal and Professor of Moral Science; his wife M.A., and Professor of everything else, and proceeded to grant degrees to all and sundry. Without insinuating that the medical schools of America are such shaky concerns as this, it may be said that several of them are still inadequate to the task of turning out competent medical men. The deficiency is apparent even in the entrance examination. Only the Bellevue Hospital, New York, has anything really equivalent to the preliminary examination of British medical schools, and that is a special matriculation examination for such students as wish to present their diplomas for recognition in Great Britain. The average demand of the medical schools from the intending student is "a good English education." This is a vague phrase, any may mean much or little, but usually any familiaritd with mathematics, physics, or natural philosophy, and even English composition, is particularly specified when required. Very few students who matriculate have already taken any degree in arts, even in Universities where there is an arts course, and in medical schools not attached to a University an arts degree is even rarer. Yet when we see that in Harvard 43 per cent. in Yale 30 per cent., and in the Columbia College 36 per cent. of the medical students have a degree in science or letters before commencing their special studies, we feel that the old country could afford to take a lesson from these places. On the other hand, in the Chicago Medical College, in connection with the North-Western University, only 3 per cent. of the students are graduates. It is to be noted, too, that the percentage of graduates is rather falling than rising. In 1881, 17 out of every 100 matriculates at the medical schools had an arts degree; in 1890 the average was only 15 per cent.

Yet a large majority of the authorities of the schools report that their present students are better educated men than their predecessors of ten years ago. Two explanations of this phenomenon may be offered. In the first place a larger number of the medical schools have each a preliminary examination. In 1880 out of 80 schools questioned on the subject, it appeared that only ten had any entrance examination at all. There would then be a larger proportion of comparatively illiterate students, who would tend to depreciate the average education of all; for, as the strength of a chain is that of its weakest link, the general receptivity of a class is something not much above that of its least intelligent members. Secondly, an improvement in the common-school education, such as has undoubtedly taken place, would tend to lessen the number of graduates in arts. For if a youth can get at school the ordinary requisites of a liberal education, he will not often, before beginning the studies for his life-work, spend time at a University in order to obtain a degree, which will be in the end a useless ornament. As a result of this, it is noted that students enter college at an earlier age than they did ten years ago; as the secretary of one college puts it, "age diminished; mental calibre advanced."

It is nevertheless admitted that the requirements for entering medical study in America are decidedly lower than they are in Britain, Germany, or France; and in the medical curriculum itself the inferiority is even more marked. The Deans of several medical schools recommend, in answer to questions from the Education Bureau, the increase of the duration of the course of study from two to three years, and of the session from five to six or eight months. Take this at its worst—two years, with a five months' session each—and we get ten months. In ten months what mortal man can learn how to treat all the ills that flesh is heir to, or even a moderate proportion of them?

We do not wish to imply that this is the average qualification of the American doctor, but, regarded even as a possible minimum, it is much to be regretted. We must note, however, that the very poverty of the training in many schools has incited to the creation and development of those post-graduate schools which give such an admirable training in special subjects. With the kind of instruction given also much fault can be found. To a large extent the instruction is not graded. A professor has a single set of lectures, which he repeats to the same students for two years in succession. This system has probably arisen from the lack of receptive faculty found in the half-educated students. Drs. Haeckel, of Jena, and Garucke, of Leipzig, agree in stating that even for scientific pursuits there is no better training than a sound classical education. Dr. Garucke admits that this result is mysterious, but finds his experience confirm it. It seems, then, that the better educated a man is when he enters medicine the more easily will he acquire a knowledge of it. The better-trained mind proves itself superior both in perception and deduction, and while the teacher's task is lightened the student's opportunities are increased.

Therefore, with the development of an increasingly high standard for matriculation there has grown an increasing attention to the practical side of medical study. "It is universally admitted," says the Dean of Tulane University Medical Department, "that, without abundant anatomical and clinical material, no medical school, however numerous and eloquent its professors, can possibly fit its pupils for practical professional life." Therefore every effort is being made in providing hospitals and anatomical museums. At the Columbia College there is a "bone-room," from which bones can be lent to students. This has proved very useful, for poor students cannot afford a large expenditure on bones, nor can they learn without them. Thence arise thefts from the dissecting-rooms, and mysterious burials and resurrections in the back garden. It is only after making sufficient provision for the student's needs that it is fair to demand much from his knowledge. There is no use in laying out a long curriculum if there is not the wherewithal, clinical and practical as well as pedagogic, to fill it up profitably. This takes time, though in America less time than elsewhere. The development of medical schools is rapidly going on. Another decade will, in all probability, see the abolition of that Southern University which holds that "an intelligent and industrious student" can acquire the whole long art of medicine in a year, and a fair proportion of the instruction now given in post-graduate courses will be made necessary to graduation.

